# Determinants of Quality of Life and Satisfaction with Life in Women with Polycystic Ovary Syndrome

**DOI:** 10.3390/ijerph15020376

**Published:** 2018-02-22

**Authors:** Ewa Rzońca, Agnieszka Bień, Artur Wdowiak, Ryszard Szymański, Grażyna Iwanowicz-Palus

**Affiliations:** 1 Faculty of Health Sciences, Medical University of Lublin, 4-6 Staszica St., 20-081 Lublin, Poland; agnesmbien@gmail.com (A.B.) spupalus@gmail.com (G.I.-P.); 2 Diagnostic Techniques Unit, Medical University of Lublin, 4-6 Staszica St., 20-081 Lublin, Poland; wdowiakartur@gmail.com; 3 Gynecological-obstetrics Ward, Independent Public Complex of Health Care Facilities in Nowa Dęba, 1a M.C. Skłodowska St., 39-460 Nowa Dęba, Poland; rsz18@poczta.fm

**Keywords:** polycystic ovary syndrome, quality of life, satisfaction, life

## Abstract

The purpose of the study was to assess the quality of life (QoL) and satisfaction with life (SwL) of women with polycystic ovary syndrome (PCOS) in comparison with those of healthy controls, and to identify and analyze factors determining QoL and SwL in women with PCOS. The cross-sectional study was performed between January and November 2016 in 504 women using health care services in Poland. The study group comprised women with PCOS, the control group women without PCOS. The study used a diagnostic survey with questionnaires. Research instruments included the World Health Organization Quality of Life-BREF (WHOQOL-BREF) questionnaire, the satisfaction with life scale (SWLS), and a standardized interview questionnaire comprising questions on the participants’ characteristics. Women with PCOS have lower QoL and SwL than healthy controls (*p* < 0.05). Factors affecting QoL in PCOS patients included socio-economic standing, time from PCOS diagnosis, BMI, age, and professional activity (*p* < 0.05). Factors affecting SwL in PCOS patients included socio-economic standing, having children, BMI, and time from PCOS diagnosis (*p* < 0.05). The higher the PCOS patients’ QoL, the higher their SwL (*p* < 0.05). Further studies are required, focusing both on PCOS and its etiology, and on its impact on the women diagnosed with the disease.

## 1. Introduction

Polycystic ovary syndrome (PCOS) is one of the most common endocrinopathies in women of reproductive age. Its etiology is not yet fully understood [1,2,3]. In accordance with the Rotterdam criteria, PCOS is diagnosed if two out of the following three findings are present: oligomenorrhea or oligo-ovulation, clinical or biochemical hyperandrogenism, and polycystic ovaries on ultrasound [1,2,4]. Women with PCOS are at risk of endometrial cancer, infertility, type 2 diabetes, and cardiovascular disease, among others. Typical PCOS symptoms include oligomenorrhea, hirsutism, obesity, acne, fertility disorders, and emotional or psychological disorders. These symptoms significantly affect the lives of PCOS patients, and in particular their psychological and emotional state, self-perception, quality of life and satisfaction with life [1,2,3,4,5,6,7,8].

Quality of life (QoL) is a broad and multidimensional concept. It is defined as an individual’s perception of their own life in the context of their culture and value systems, and their personal goals, standards and concerns [9,10,11]. Studies on the QoL of individuals diagnosed with chronic illnesses are of interest to health care professionals. They allow them to learn about the subjective impact of the disease on the patients’ life and wellbeing, as well as its impact on care and the diagnostic and therapeutic interventions made [9,11]. In turn, satisfaction with life (SwL) is a significant component of subjective wellbeing [9,12,13]. SwL is established by making a comparison between one’s situation on the one hand, and one’s standards and goals on the other [13,14,15]. Therefore, both QoL and SwL are evaluated and perceived in a unique, individual way by each person, and determined by multiple factors [12,14].

The purpose of the study was to assess the QoL and SwL of women with PCOS in comparison with those of healthy controls. The authors also attempted to identify and analyze socio-demographic factors determining QoL and SwL in women with PCOS.

## 2. Materials and Methods

### 2.1. Subjects

The cross-sectional study was performed between January and November 2016 in 504 women using health care services (primary care, specialist outpatient care, and inpatient/hospital care) in four regions of Poland: Lublin, Podkarpacie, Pomerania, and Greater Poland Provinces. Inclusion criteria for cases were as follows: age over 18 years, PCOS diagnosis made based on the Rotterdam criteria, and use of health care services in Poland. Due to the research scope, the sample was selected in a targeted rather than probabilistic manner. Exclusion criteria for the study group were cancer diagnosis and psychiatric disorders. Two hundred and fifty correctly completed questionnaires were returned in the study group. The survey response rate was 83.33%. Inclusion criteria for controls were as follows: age over 18 years, use of health care services in Poland, and no PCOS diagnosis. Exclusion criteria for the control group were cancer diagnosis, chronic illness, and psychiatric disorders. Two hundred and fifty-four correctly completed questionnaires were returned in the control group—the survey response rate was 84.67%. The study was performed in accordance with the Helsinki Declaration, and approved by the Lublin Medical University Bioethics Committee (approval NO. KE-0254/189/2015). It was also approved by the respective health care units. Respondents were informed that participation was voluntary, and that study results were anonymous and to be used exclusively for research purposes.

### 2.2. Assessments

The study used a diagnostic survey with questionnaires. Research instruments included the World Health Organization Quality of Life-BREF (WHOQOL-BREF) questionnaire (in Polish), the satisfaction with life scale (SWLS), and a standardized interview questionnaire comprising questions on the participants’ characteristics.

The WHOQOL-BREF is a short version of the WHO Quality of Life Questionnaire, assessing the QoL of ill or healthy individuals. It comprises 26 items assessing overall perceived QoL, overall perceived health, and Quality of Life in four specific domains: physical, psychological, social, and environmental. Scores in each domain are determined by calculating means from all items contained in a given domain. For each domain, the score is between 4 and 20 points, with higher scores denoting higher QoL. The questionnaire was adapted for Polish settings by Jaracz and Wołowicka. The questionnaire reliability, measured by the internal consistency coefficient (Cronbach’s α), is 0.54–0.91 for individual domains; for the whole scale it is 0.92 in healthy individuals and 0.95 in ill individuals [16,17,18]. 

The SWLS was developed by Diener et al. and adapted for Polish settings by Juczyński. It measures SwL, both in healthy and ill individuals, through a comparison between their living conditions and their own standards. The scale comprises five statements, scored between 1 and 7, with 1 standing for “strongly disagree”, and 7 standing for “strongly agree”. The total score is the sum of points for all items, between 5 and 35 points, with higher scores denoting more satisfaction with life. The raw score is converted to a (10-point) sten scale, with results between sten 1 and 4 considered low, sten 5–6 considered moderate, and 7–10 considered high. Questionnaire reliability as measured by Cronbach’s α is 0.82 [19].

### 2.3. Statistical Analyses

Statistical analysis was performed for the results obtained. The software used for databases and statistical analysis was STATISTICA version 12 (StatSoft, Cracow, Poland). Quantitative data were presented using means, standard deviations, and median; qualitative data were presented using numbers and percentages.

Variable distribution in the groups studied was tested using the Shapiro–Wilk test for normality. Differences between two groups were tested using the parametric Student’s *t* test and non-parametric Mann–Whitney U test; for comparing more than two groups, the Kruskal–Wallis test was used. The effect size was calculated with Cohen’s *d* and Glass’s *delta*. Correlations between the variables analysed were tested with a chi-squared test (χ^2^). To evaluate variables effecting the strength of the WHOQOL-BREF and SWLS variables, standard multiple regression analysis was performed. With this method, interpretation of results involves comparing values of the *β* coefficient, which is interpreted with regard to the strength and direction of the relationship between individual variables. The adjusted *R*^2^ value shows how well the regression model fits the data. Reference categories were provided for nominal and ordinal variables: for age, it was under 25 y/o, for having children—no children, and for time from PCOS diagnosis—less than 1 year. BMI was included in the analysis as a scale variable. If a variable comprised two categories, the analysis involved comparing the two categories, e.g., in the case of professional activity, it was working professionally and socio-economic status—unsatisfactory. Correlations between selected variables were tested using Pearson’s *r,* correlation strength was evaluated using Guilford’s classification. All tests used in the study had high statistical power: 98–100% for regression analysis, 99–100% for WHOQOL-BREF score comparisons using Student’s *t*-test, and 85.1% for analysis of differences in SWLS scores between groups. The study used a significance level of *p* < 0.05.

## 3. Results

Table 1 shows the participants’ characteristics. Most cases were women aged 26–35 years (43.20%), professionally active (77.20%), who viewed their socio-economic standing as satisfactory (62.40%), had no children (53.20%), had normal BMI (41.20%), and had been diagnosed with PCOS 1 to 5 years before (50.40%). As to the controls, most were women aged 26–35 years (44.09%), professionally active (71.65%), who viewed their socio-economic standing as satisfactory (67.72%), had two or more children (43.70%), and had normal BMI (61.42%).

Patients in the study group had a lower overall QoL (*p* = 0.000), worse perceived health (*p* = 0.000), and lower QoL in all specific domains: physical (*p* = 0.023), psychological (*p* = 0.000), environmental (*p* = 0.000), and social (*p* = 0.000), compared with controls (Table 2).

Table 3 presents the regression model for the WHOQOL-BREF variable with regard to overall QoL and perceived health. Socio-economic standing (*β* = 0.375; *p* = 0.000) and time from PCOS diagnosis—1–5 years (*β* = −0.182; *p* = 0.044) and 6–10 years (*β* = −0.175; *p* = 0.045)—proved to be statistically significant variables for overall QoL in the WHOQOL-BREF regression model. The better the socio-economic standing, the higher the overall QoL, and the longer the time from PCOS diagnosis (1–5 years; 6–10 years), the lower the overall QoL. On the other hand, statistically significant variables of overall perceived health in the WHOQOL-BREF regression model were professional activity (*β* = −0.137; *p* = 0.035), socio-economic standing (*β* = 0.244; *p* = 0.000) and time from PCOS diagnosis—6–10 years (*β* = −0.183; *p* = 0.047). Lack of professional activity and long time from PCOS diagnosis (6–10 years) translate into worse overall perception of health. Conversely, the better the socio-economic standing, the better the overall perception of health.

Table 4 presents the regression model for the WHOQOL-BREF variable with regard to physical, psychological, social and environmental domains. Socio-economic standing (*β* = 0.311; *p* = 0.000) and time from PCOS diagnosis—1–5 years (*β* = −0.183; *p* = 0.047) proved to be statistically significant variables for the physical domain in the WHOQOL-BREF variable model. The better the socio-economic standing, the higher the QoL in the domain mentioned, and the longer the time since PCOS diagnosis (1–5 years), the lower the QoL in this domain. Statistically significant factors related for the psychological domain in the WHOQOL-BREF variable model were socio-economic standing (*β* = 0.302; *p* = 0.000) and BMI (*β* = –0.192; *p* = 0.002). The better the socio-economic standing, the higher the QoL in the psychological domain, and the higher the BMI index, the lower the QoL in this domain. Statistically significant variables for the social domain in the WHOQOL-BREF regression model were age (*β* = –0.214; *p* = 0.049), socio-economic standing (*β* = 0.355; *p* = 0.000) and time from PCOS diagnosis—1–5 years (*β* = –0.261; *p* = 0.004) and 6–10 years (*β* = –0.199; *p* = 0.023). The better the socio-economic standing, the higher the QoL in the social domain, and the older the age and longer the time from PCOS diagnosis (1–5 years; 6–10 years), the lower the QoL in this domain. Statistically significant variables for the environmental domain in the WHOQOL-BREF regression model were: socio-economic standing (*β* = 0.462; *p* = 0.000), BMI (*β* = –0.122; *p* = 0.030) and time from PCOS diagnosis—1–5 years (*β* = –0.229; *p* = 0.007) and 6–10 years (*β* = –0.190; *p* = 0.020). The better the socio-economic standing, the higher the QoL in the environmental domain, and the higher the BMI index and longer the time from PCOS diagnosis (1–5 years; 6–10 years), the lower the QoL in this domain.

Comparison of SwL showed that respondents with PCOS were less satisfied with life than those without the syndrome (*p* = 0.002)—Table 5.

Table 6 presents the regression model for the SWLS variable. Statistically significant factors related in this model were: socio-economic standing (*β* = 0.380; *p* = 0.000), experience of motherhood—having one child (*β* = 0.158, *p* = 0.016)—BMI (*β* = –0.121; *p* = 0.035) and time from PCOS diagnosis—1–5 years (*β* = –0.206; *p* = 0.017) and 6–10 years (*β* = –0.261; *p* = 0.002). The better the socio-economic standing, the higher the SWLS score. Also, having one child translated into a higher SWLS score. On the other hand, the higher the BMI index and longer the time from PCOS diagnosis (1–5 years; 6–10 years), the lower the satisfaction with life. 

Table 7 shows correlations between the women’s QoL and SwL. QoL and SwL were found to be significantly positively correlated in PCOS patients (*p* = 0.000). The correlations were rated at between 0.447 and 0.583.

## 4. Discussion

Women’s health and its determinants are the subject of numerous studies worldwide [2,5,8,10,11,13,14]. PCOS is a disease with a complex symptomatology and diverse clinical presentations, which significantly affects the patients’ life and functioning [2,3,8]. Research shows that PCOS adversely affects women’s quality of life, wellbeing, self-esteem, psychological status, and body image [3,20,21,22,23,24]. The purpose of this paper was to assess the quality of life and satisfaction with life of women with PCOS, as well as their determinants.

Studies on PCOS patients’ QoL worldwide showed that these women have lower QoL than healthy controls, which is also corroborated by the present study [23,25,26,27]. Polish respondents obtained the highest QoL scores in the social domain, and the lowest in the physical domain. In a study by Benetti-Pinto et al., Brazilian PCOS patients perceived their QoL as the highest in the physical domain, and the lowest in the environmental domain [25]. Women from South Asia participating in a study by Kumarapeli et al. scored highest in the psychological QoL domain, and lowest in the social domain [26]. Shafti & Shahbazi studied Iranian women with PCOS, who had the highest QoL in the environmental domain, and the lowest in the social domain [21]. The variability in broadly understood quality of life, discussed above, may be associated with the subjective experience of the multiple PCOS symptoms and their intensity. Health-related quality of life (HRQoL) in women with PCOS has also been studied worldwide. In these studies, decreased HRQoL in PCOS patients was shown to be associated with menstrual disorders, difficulty maintaining normal body weight, hirsutism, impaired fertility, or emotional disorders, i.e., with symptoms produced by the syndrome [8,28,29,30]. The subjectively perceived impact of PCOS symptoms on the women’s lives, as well as cultural considerations or the women’s expectations that may be restricted by the syndrome (e.g., the desire to have children in the case of fertility problems), provide a basis for a better understanding of this large variability in broadly understood quality of life in the physical, psychological, environmental, and social domains [20,22,24,28,29,30].

Studies on factors affecting QoL in patients with chronic illnesses demonstrate a multitude of diverse determinants [11,31,32,33,34]. In their study on the determinants of QoL in diabetes patients, Didarloo & Alizadeh reported that age, duration of illness, education, monthly income, and the presence of comorbidities were all significantly associated with QoL in women with type 2 diabetes [11]. Bień et al. demonstrated that the QoL of pregnant women with diabetes was affected by their financial standing, perceived health, knowledge on the disease, restrictions in daily life, and treatment used [31]. Chrobak-Bień et al. studied the determinants of QoL in patients with Crohn’s disease, and reported the intensity of disease symptoms experienced, as well as patient age and education, as significant [32]. In a study by Bąk-Drabik & Zior, involving patients with chronic obstructive pulmonary disease, QoL was determined by spirometric abnormalities, and the patients’ education, monthly income, profession, and employment [33]. Lemos et al. studied patients with chronic kidney disease, finding QoL to be determined by the patients’ sex, age, and most of all, family income [34]. The authors’ own analyses of QoL in PCOS patients showed that patients who subjectively viewed their socio-economic standing as satisfactory had higher overall QoL. Compared to professionally active respondents, women who lacked this type of activity had a lower overall perception of health. Another factor significantly affecting QoL in PCOS patients was age, as QoL in the social domain decreased in older patients. Notably, research on women’s QoL demonstrates that it decreases with age, as in the study by Salehi et al. on self-assessed quality of life and the lifestyle of young Iranian women [10]. The aspect of an age-related QoL decrease is particularly emphasized in studies on menopausal women [35,36,37]. Like the present study, an association between higher BMI and lower QoL was reported by Esenbruch et al. [27], Hanh et al. [23], Kumarapeli et al. [26], and Benetti-Pinto et al. [25]. Time from PCOS diagnosis was also a significant determinant of QoL in the women with PCOS. The longer the illness duration, the lower the overall QoL, perception of health, and QoL in the physical, social, and environmental domains. Didarloo & Alizadeh also emphasized the decrease in QoL in women with type 2 diabetes seen with longer disease [11].

In addition to QoL, satisfaction with life is another significant component in the assessment of an individual’s life [12,13,38,39]. It is perceived and valued individually by each person, based on a unique, personal set of criteria, and long-term reflection [12,14,38,39,40]. The satisfaction with life scale is one of the most commonly used instruments for assessing SwL in both healthy and ill individuals. It was developed by Diener et al. [12,41,42], and adapted for Polish settings by Juczyński [19]. The author of its Polish adaptation reported mean scores for Polish populations, including a population of healthy women: 21.09; diabetics: 20.34; or women experiencing pregnancy complications: 21.34 [19]. The mean SwL score in young Iranian women was reported as 19.9 [12], and in infertile Iranian women—23.65 [41]. Gebuza et al. (2014) found women in the third trimester of pregnancy to have a mean SwL score of 24.51, and postpartum women—5.71 [42]. A comparison of the reported means with the mean score found for PCOS patients in the present study (20.16) warrants the conclusion that the women with PCOS were less satisfied with life than the other groups studied. The SWLS allows for expressing an individual’s satisfaction with their own personal achievements and with their living conditions. Low satisfaction with life is found when there is a significant disparity between expectations and actual results [19,42]. Such a disparity was found in the present study—women with PCOS were less satisfied with life than healthy controls. A study by Elsenbruch et al. (2003), focusing on various aspects of life in women with PCOS, demonstrated that these women had significantly lower SwL in terms of health, self-esteem, and sex life than controls [27]. Feller et al. (2013) reported that low SwL was associated with chronic illness [14]. Lukkala et al. (2016) studied SwL in post-menopausal women, and reported that the disease burden seemed to be more significant to SwL than the particular kind of disease [13], which may provide some explanation of the SwL scores in the Polish women with PCOS who were studied. 

SwL has multiple determinants, which include demographic factors, socio-economic standing, social support, self-perception, and the individual’s physical or psychological condition [12,38,39,40,42]. Harris et al. (2016) demonstrated that SwL in young Iranian women was determined by their income, living conditions, age, marital status, ethnicity, education, and religious attitudes [12]. Bień et al. (2017) studied women who were childless by choice, and demonstrated that their SwL was determined by such socio-demographic factors as age, residence, education, marital status, and financial standing [43]. Socio-economic standing, which comprises income, education, and living conditions, is a significant determinant of SwL, as reported in SwL studies by Abbott & Sapsford (2006) in Russians and Ukrainians, Jan & Masood (2008) in women, Harris et al. (2016) in young Iranian women, and Bień et al. (2017) in women who decided to remain childless [12,40,43,44]. The association between satisfactory socio-economic standing and higher SwL is also corroborated by the present study. The analysis also showed that the higher the BMI index and the longer the time from PCOS diagnosis, the lower the satisfaction with life in the women studied. These socio-demographic factors associated with SwL also affected the QoL of the PCOS patients studied. The analysis of PCOS women’s SwL also showed having children to be a significant determinant. Infertility is a very important issue in modern medicine, especially seeing as PCOS women are found to have fertility problems significantly more often. For women who want to have children, failure to conceive may cause a life crisis, which decreases SwL [8,15,45]. The authors’ own research indicated that the experience of motherhood, i.e., having a child, translates into higher satisfaction with life in the women with PCOS participating in the study.

The authors’ own analyses demonstrated an association between higher overall QoL, better perceived health, and higher QoL in the specific domains on the one hand, and the PCOS patients’ SwL on the other. In their study on self-reported QoL and its associations with lifestyle in young Iranian women, Salehi et al. (2015) also reported a positive correlation between all QoL domains and SwL [10]. 

PCOS is a syndrome that adversely affects physical, psychological, and social functioning, overall QoL, and SwL, as clearly evidenced both by the present study and by other studies worldwide [21,25,26,27]. Considering the impact of PCOS diagnosis on women’s quality of life, functioning and experiences, the patients’ condition and needs must be studied in order to prioritize care appropriately. Enhancement of care and education may improve self-care in these women, also contributing to better perceived QoL and SwL. Understanding of PCOS patients’ perceived QoL may help optimize care provided by health care professionals, improving its quality and better fulfilling the patients’ expectations. Therefore, further studies are required, focusing both on PCOS and its etiology, and on its impact on the women diagnosed with the disease.

The study has certain limitations. The study only includes an analysis and evaluation of socio-demographic factors that affect the studied women’s QoL based on the WHOQOL-BREF questionnaire, i.e., overall QoL, overall perceived health, and QoL in specific domains: physical, psychological, social, and environmental. The study concept did not involve analyzing the impact of PCOS symptoms or the patients’ psychological condition on their life and functioning. Therefore, the study does not analyze and evaluate health-related quality of life, i.e., QoL affected by PCOS symptoms, e.g. hirsutism or menstrual disorders.

## 5. Conclusions

Women with PCOS have lower QoL and SwL than healthy ones. Factors affecting QoL in PCOS patients include socio-economic standing, time from PCOS diagnosis, BMI, age, and professional activity. Factors affecting SwL in PCOS patients include socio-economic standing, having children, BMI, and time from PCOS diagnosis. The higher the PCOS patients’ QoL, the higher their SwL.

## Figures and Tables

**Table 1:** Participants’ characteristics.

Participants’ Characteristics		Study Group	Control Group	Statistical Analysis
		n (%)	n (%)	
		250 (49.60)	254 (50.40)	
Age	Under 25 years old	47 (18.80)	42 (16.54)	χ^2^ = 0.450 *p* = 0.798
	26–35	108 (43.20)	112 (44.09)	
	Over 35 years old	95 (38.00)	100 (39.37)	
Professional activity	Working professionally	193 (77.20)	182 (71.65)	χ^2^ = 2.035 *p* = 0.153
	Not working	57 (22.80)	72 (28.35)	
Socio-economic standing	Unsatisfactory	94 (37.60)	82 (32.28)	χ^2^ = 1.567 *p* = 0.210
	Satisfactory	156 (62.40)	172 (67.72)	
Having children	No children	133 (53.20)	80 (31.50)	χ^2^ = 28.668 *p* = 0.000
	One child	57 (22.80)	63 (24.80)	
	Two or more children	60 (24.00)	111 (43.70)	
BMI	Normal (18.50–24.99)	103 (41.20)	156 (61.42)	χ^2^ = 24.535 *p* = 0.000
	Overweight (25.00–29.99)	98 (39.20)	77 (30.31)	
	Obese (>30.00)	49 (19.60)	21 (8.27)	
	Mean BMI	26.41	24.33	
Time from PCOS diagnosis	Up to 1 year	38 (15.20)	---	---
	1–5 years	126 (50.40)	---	
	6–10 years	52 (20.80)	---	
	more than 10 years	34 (13.60)	---	

BMI—Body Mass Index; PCOS—Polycystic ovary syndrome.

**Table 2:** Comparison of QoL World Health Organization Quality of Life-BREF (WHOQOL-BREF scores) between the study and control groups.

WHOQOL-BREF—Domains	Study Group			Control Group			Statistical Analysis
	M	SD	Me	M	SD	Me	
General quality of life	3.72	0.77	4.00	4.01	0.60	4.00	Z = −4.446, *p* = 0.000, *r_c_* = 0.198
General health	3.28	0.91	3.00	3.72	0.73	4.00	Z = −5.669, *p* = 0.000, *r_c_* = 0.263
Physical health	53.88	11.87	56.00	56.02	9.11	56.00	*t* = −2.276, *p* = 0.023, *d* = 0.202
Psychological	59.88	15.09	63.00	66.23	10.27	69.00	*t* = −5.512, *p* = 0.000, *d* = 0.492
Social relationships	67.12	18.18	69.00	74.37	15.90	75.00	*t* = −4.867, *p* = 0.000, *d* = 0.424
Environment	63.35	14.90	63.00	70.21	12.61	69.00	*t* = −5.584, *p* = 0.000, *d* = 0.497

M—mean; SD—standard deviation; Me—median.

**Table 3:** Regression model for the WHOQOL-BREF variable—general quality of life and general health.

Variables	General Quality of Life				General Health			
	*R*^2^ = 0.176, *F*(12.237) = 5.425, *p <* 0.001				*R*^2^ = 0.081, *F*(12.237) = 2.833, *p =* 0.001			
	*B*	*β*	*t*	*p*	*B*	*β*	*t*	*p*
Age: 26–35	0.000	0.000	−0.002	0.999	0.079	0.043	*0.457*	0.648
Age: over 35 years old	−0.266	−0.168	−1.530	0.127	0.087	0.047	0.406	0.685
Professional activity (non-working)	−0.112	−0.061	−1.004	0.316	−0.294	−0.137	−2.126	0.035
Socio–economic standing (satisfactory)	0.596	0.375	6.208	0.000	0.456	0.244	3.827	0.000
Having children: one child	−0.110	−0.060	−0.865	0.388	0.148	0.069	0.942	0.347
Having children: two or more children	0.213	0.118	1.383	0.168	0.317	0.150	1.659	0.098
BMI	−0.014	−0.086	−1.439	0.152	−0.003	−0.015	−0.241	0.810
Time from PCOS diagnosis: 1–5 years	−0.280	−0.182	−2.021	0.044	−0.314	−0.173	−1.825	0.069
Time from PCOS diagnosis: 6–10 years	−0.333	–0.175	–2.015	0.045	–0.408	–0.183	–1.995	0.047
Time from PCOS diagnosis: more than 10 years	−0.095	−0.042	−0.487	0.626	−0.338	−0.128	−1.393	0.165

BMI—Body Mass Index; PCOS—Polycystic ovary syndrome.

**Table 4:** Regression model for the WHOQOL-BREF variable—domains.

Variables	Physical Health				Psychological				Social Relationships				Environment			
	*R*^2^ = 0.143, *F*(12.237) = 4.460, *p <* 0.001				*R*^2^ = 0.167, *F*(12.237) = 5.166, *p <* 0.001				*R*^2^ = 0.174, *F*(12.237) = 5.365, *p <* 0.001				*R*^2^ = 0.285, *F*(12.237) = 9.262, *p <* 0.001			
	*B*	*β*	*t*	*p*	*B*	*β*	*t*	*p*	*B*	*β*	*t*	*p*	*B*	*β*	*t*	*p*
Age: 26–35	2.769	0.088	0.958	0.339	1.790	0.059	0.649	0.517	–0.674	–0.018	–0.204	0.839	1.371	0.046	0.544	0.587
Age: over 35 y/o	–1.867	–0.058	–0.521	0.603	–2.512	–0.081	–0.736	0.463	–8.004	–0.214	–1.953	0.049	–1.222	–0.040	–0.391	0.696
Professional activity (non-working)	–2.705	–0.073	–1.175	0.241	–3.127	–0.087	–1.424	0.156	–2.079	–0.048	–0.789	0.431	–0.146	–0.004	–0.073	0.942
Socio–economic standing (satisfactory)	9.987	0.311	5.045	0.000	9.404	0.302	4.981	0.000	13.283	0.355	5.863	0.000	14.180	0.462	8.208	0.000
Having children: one child	0.074	0.002	0.028	0.978	3.731	0.104	1.496	0.136	2.219	0.051	0.741	0.459	–0.961	–0.027	–0.421	0.674
Having children: two or more children	0.0058	0.002	0.018	0.985	3.975	0.113	1.312	0.191	5.613	0.132	1.544	0.124	–1.010	–0.029	–0.364	0.716
BMI	–0.397	–0.118	–1.930	0.055	–0.624	–0.192	–3.184	0.002	–0.242	–0.062	–1.027	0.305	–0.392	–0.122	–2.182	0.030
Time from PCOS diagnosis: 1–5 years	–5.698	–0.183	–1.995	0.047	–2.854	–0.095	–1.048	0.296	–9.476	–0.261	–2.899	0.004	–6.811	–0.229	–2.732	0.007
Time from PCOS diagnosis: 6–10 years	–6.503	–0.170	–1.911	0.057	–4.210	–0.113	–1.297	0.196	–8.904	–0.199	–2.286	0.023	–6.978	–0.190	–2.349	0.020
Time from PCOS diagnosis: more than 10 years	–2.726	–0.060	–0.676	0.500	2.033	0.046	0.529	0.598	–2.407	–0.045	–0.522	0.602	0.135	0.003	0.038	0.969

BMI—Body Mass Index; PCOS—Polycystic ovary syndrome.

**Table 5:** Comparison of satisfaction with life (SwL) (satisfaction with life scale (SWLS) scores) between the study and control groups.

Satisfaction with Life	Study Group			Control Group			Statistical Analysis
	M	SD	Me	M	SD	Me	
SWLS	20.16	6.25	21.00	21.74	5.55	22.00	*t* = –3.014, *p* = 0.002, *d* = 0.098

M—mean; SD—standard deviation; Me—median.

**Table 6:** Regression model for the SWLS variable.

Variables	SWLS			
	*R*^2^ = 0.259, *F*(12.237) = 8.269, *p <* 0.001			
	*B*	*β*	*t*	*p*
Age: 26–35	1.948	0.155	1.809	0.072
Age: over 35 y/o	–0.047	–0.004	–0.035	0.972
Professional activity (non-working)	–1.543	–0.104	–1.799	0.073
Socio–economic standing (satisfactory)	4.891	0.380	6.632	0.000
Having children: one child	2.355	0.158	2.417	0.016
Having children: two or more children	1.277	0.087	1.079	0.282
BMI	–0.163	–0.121	–2.125	0.035
Time from PCOS diagnosis: 1–5 years	–2.569	–0.206	–2.414	0.017
Time from PCOS diagnosis: 6–10 years	–4.019	–0.261	–3.170	0.002
Time from PCOS diagnosis: more than 10 years	–0.042	–0.002	–0.028	0.978

BMI—Body Mass Index; PCOS—Polycystic ovary syndrome.

**Table 7:** Correlations between the women’s QoL (WHOQOL-BREF scores) and SwL (SWLS scores).

WHOQOL-BREF—Domains	SWLS	
	*r*	*p*
General quality of life	0.496	0.000
General health	0.531	0.000
Physical health	0.447	0.000
Psychological	0.575	0.000
Social relationships	0.581	0.000
Environment	0.583	0.000
